# The adverse characteristics of hepatocellular carcinoma in the non‐cirrhotic liver disproportionately disadvantage Black patients

**DOI:** 10.1002/cam4.6654

**Published:** 2024-01-17

**Authors:** Tali Shaltiel, Umut Sarpel, Andrea D. Branch

**Affiliations:** ^1^ Division of Surgical Oncology, Department of Surgery Icahn School of Medicine at Mount Sinai New York New York USA; ^2^ Division of Liver Diseases, Department of Medicine Icahn School of Medicine at Mount Sinai New York New York USA

**Keywords:** African American, Black, disparities, equity, Fibrosis‐4, hepatocellular carcinoma, insurance, Milan criteria, surveillance

## Abstract

**Background:**

Black patients have higher hepatocellular carcinoma (HCC)‐related mortality than White patients and more often develop HCC in non‐cirrhotic liver. HCC surveillance is primarily directed toward cirrhotic patients. We aimed to characterize HCC in non‐cirrhotic patients and to identify factors associated with HCC beyond Milan criteria.

**Methods:**

Demographic, imaging, laboratory, and pathology data of HCC patients at our institution, 2003–2018, were reviewed, retrospectively. Race/ethnicity were self‐reported. Cirrhosis was defined as a Fibrosis‐4 score ≥3.25.

**Results:**

Compared to 1146 cirrhotic patients, 411 non‐cirrhotic patients had larger tumors (median 4.7 cm vs. 3.1 cm, *p* < 0.01) and were less likely to be within Milan criteria (42.6% vs. 57.7%, *p* < 0.01). Among non‐cirrhotic patients, Black patients had larger tumors (4.9 cm vs. 4.3 cm, *p* < 0.01) and a higher percentage of poorly differentiated tumors (39.4% vs. 23.1%, *p* = 0.02). Among cirrhotic patients, Black patients had larger tumors (3.3 cm vs. 3.0 cm, *p* = 0.03) and were less likely to be within Milan criteria (52.3% vs. 83.2%, *p* < 0.01). In multivariable analysis, lack of commercial insurance (OR 1.45 [CI 95% 1.19–1.83], *p* < 0.01), male sex (OR 1.34 [CI 95% 1.05–1.70], *p* < 0.01), absence of cirrhosis (OR 1.58 [CI 95% 1.27–1.98], *p* < 0.01) and Black race/ethnicity (OR 1.34 [CI 95% 1.09–1.66], *p* = 0.01) were associated with HCC beyond Milan criteria. Black patients had lower survival rates than other patients (*p* < 0.01).

**Conclusions:**

Non‐cirrhotic patients had more advanced HCC than cirrhotic patients. Black patients (with or without cirrhosis) had more advanced HCC than comparable non‐Black patients and higher mortality rates. Improved access to healthcare (commercial insurance) may increase early diagnosis (within Milan criteria) and reduce disparities.

## 1 INTRODUCTION

Hepatocellular carcinoma (HCC) is the predominant histological type of primary liver cancer. HCC is the third most common cause of site‐specific cancer‐related death world‐wide.^1^, ^2^ In 2018, the HCC‐mortality rate in the United States was 4.8 per 100,000 person‐years.^3^ The United States is expected to have over 56,000 newly diagnosed cases of HCC in 2030.^4^ HCC can often be cured by surgical resection or liver transplantation if it is detected at a sufficiently early stage. Unfortunately, most cases are too advanced at diagnosis for surgical cure,^5^ and 1‐year survival is below 60%.^6^ There are ongoing efforts to develop and implement more effective surveillance programs to increase early detection and survival.

HCC is nearly always associated with an underlying chronic liver disease. Hepatitis C virus (HCV) infection is the leading risk factor for HCC in the United States and Western Europe.^7^, ^8^ Alcohol‐related liver disease (ALD), hepatitis B virus (HBV) infection, smoking, toxic exposures, and metabolic dysfunction‐associated steatotic liver disease (MASLD) are also important underlying diseases.^9^ Cirrhosis precedes HCC development in up to 90% of cases.^10^ Practice guidelines recommend HCC surveillance for all patients with cirrhosis, irrespective of etiology. Recommendations for surveillance of non‐cirrhotic patients are more complex. The American Association for the Study of Liver Disease (AASLD)^11^, ^12^ and the Asian Pacific Association for the Study of Liver Disease (APASL)^13^ recommend surveillance for some non‐cirrhotic patients with chronic HBV infection. The European Association for the Study of Liver Disease (EASL)^14^ and Japanese Society of Hepatology^15^ also recommend risk factor‐based screening for selected non‐cirrhotic patients.

Significant racial disparities exist in the presentation, treatment, and prognosis of HCC.^16^, ^17^ Black patients with newly diagnosed HCC have larger and more advanced tumors and are more likely to have poorly differentiated tumors.^18^ Despite more advanced HCC, liver disease is less advanced in Black patients with HCV infection^18^, ^19^, ^20^, ^21^ and Black patients with HBV exposure are more likely to develop HCC in the absence of cirrhosis than White patients.^22^, ^23^ These findings are consistent with evidence that non‐Hispanic Black patients have slower fibrosis progression than White or Hispanic patients.^24^, ^25^ Ironically, the lack of advanced cirrhosis at the time of HCC development may disadvantage Black patients by causing them and their providers to be less vigilant about adhering to HCC surveillance guidelines.

Black patients with HCC have worse survival than White, Hispanic, or Asian patients.^16^, ^18^, ^19^, ^20^, ^21^ In the United States, Black patients are less likely to be diagnosed with early‐stage HCC than White patients.^17^ The factors leading to worse outcomes are complex and multifactorial. They may include differences in genomic risk factors, toxic and mutagenic exposures, and reduced access to healthcare.^26^, ^27^, ^28^ Blacks are more likely to have genetic polymorphisms that drive production of interferon λ‐4 than members of other racial and ethnic groups.^29^ This might predispose to aggressive HCC, similar to what occurs in prostate cancer where the high interferon λ‐4‐expressing genotype is associated with more aggressive disease and reduced survival.^30^ Additionally, Blacks have lower HCC surveillance rates than members of other racial/ethnic groups^26^ and are less likely to receive serial surveillance.^16^ Compared to White patients, Black patients are less likely to receive surveillance with more sensitive imaging modalities (CT and MRI) and more likely to receive ultrasound imaging.^31^ After diagnosis, Black patients are referred for curative therapy less frequently,^26^, ^32^ and receive less treatment than other patients.^33^ Limited insurance coverage, less HCC surveillance, atypical presentation in a liver with less advanced liver disease, genetic factors, more aggressive tumors, less intensive HCC treatment, under‐representation in clinical trials and molecular studies, and systemic racism may all contribute to greater morbidity and mortality.

Herein, we compare liver disease status and HCC characteristics in patients with and without cirrhosis, and determine the distinctive features of HCC in Black patients across all major liver disease etiologies and among Black patients who do not have HCV exposure as the underlying chronic liver disease; we previously conducted a detailed study of HCC in patients with chronic HCV exposure.^18^ We test four hypotheses: (a) that Black patients diagnosed with HCC and without HCV exposure have better liver function than non‐Black patients, but Black patients have more aggressive tumors, (b) that HCC in non‐cirrhotic patients has worse prognostic features than HCC in cirrhotic patients, (c) that across all major types of underlying liver disease, HCC in Black patients has worse prognostic features than HCC in non‐Black patients, and (d) that a higher percentage of HCC develops in non‐cirrhotic livers in Black patients than in non‐Black patients. Confirmation of these hypotheses would add significantly to literature emphasizing the urgent need for tools to risk‐stratify non‐cirrhotic patients and identify patients likely to benefit from HCC surveillance and would indicate that Black patients may need to begin HCC surveillance at an earlier fibrosis stage than non‐Black patients.

## 2 MATERIALS AND METHODS

This retrospective study was approved by the Institutional Review Board of the Icahn School of Medicine at Mount Sinai, protocol number 18–01233 with a waiver of informed consent. The records of HCC patients with HBV exposure, ALD, or NAFLD who were diagnosed with HCC at our institution from 2003 to August 2018 were collected and analyzed as a dataset. In addition, data on these patients were combined with previously published data of HCC patients with HCV infection^18^ and the combined dataset was analyzed as a whole. A preliminary report of the findings was presented previously.^34^

### 2.1 Characteristics of HCC patients with HBV, ALD or NAFLD

A preliminary list of patients was generated using the international classification of diseases (ICD)‐9 code 155.0, and cases were confirmed by manual review, applying accepted radiographic and/or pathologic criteria. Patients assigned to non‐HCV etiologies had a documented negative test for HCV antibody and did not have a positive test for HCV RNA or HCV genotype.

HBV exposure was defined by seropositivity for one or more of the following: HBV surface antigen (HBsAg), HBV envelope antigen (HBeAg), HBV core antibody (HBcAb), or HBV DNA. Asian patients with HBV were excluded because of the high likelihood that HBV was acquired by vertical transmission, which carries greater HCC risk than horizontal transmission,^35^, ^36^ the typical mode of transmission among non‐Asian patients in the United States.^37^ ALD was diagnosed by the treating hepatologist, with documentation in the medical record and/or the ICD‐9 code 571.0–3. NAFLD was diagnosed by the treating hepatologist, with documentation in the medical record and/or a related ICD‐9 code. Patients with other identified causes of liver disease (*n* = 7) were excluded.

Sociodemographic and anthropometric data about race, ethnicity, age, sex, body mass index (BMI), and type of insurance were collected. Race and ethnicity were self‐identified. Patients lacking race/ethnicity data were excluded. Patients were classified as non‐Hispanic Black or non‐Black (all others). Government‐insured patients had Medicare and no supplemental insurance or Medicaid. Commercially‐ensured patients had non‐government‐subsidized insurance or Medicare plus supplemental private insurance.

### 2.2 Laboratory variables

The clinical laboratory test results most proximate to the date of HCC diagnosis were collected and analyzed. Data included platelet count, alanine aminotransferase, aspartate aminotransferase, albumin, total bilirubin, international normalized ratio (INR), and α‐fetoprotein (AFP). Human immunodeficiency virus (HIV) infection was indicated by HIV RNA and/or anti‐HIV antibodies in serum.

Liver function and fibrosis stage were determined using the model for end stage liver disease (MELD) score,^38^ Child‐Pugh classification, and the Fibrosis‐4 (FIB‐4) index score calculated as: age (years) × aspartate amino transferase (U/L)/platelets (10^9^) × √ alanine aminotransferase (U/L).^39^ Cut‐off values of FIB‐4 vary based on liver disease etiology.^39^, ^40^, ^41^ The FIB‐4 score is predictive of HCC risk.^42^ A FIB‐4 score ≥3.25 was used to classify patients as cirrhotic in this study. The AST to Platelet Ratio Index (APRI) score, an additional non‐invasive indicator of cirrhosis and HCC risk,^43^, ^44^ was also calculated.

### 2.3 Radiologic and histopathological variables

Imaging modalities included abdominal contrast‐enhanced computerized tomography (CT) and magnetic resonance imaging (MRI). Radiologic findings at the time of HCC diagnosis were extracted from imaging reports: determinations of cirrhosis or portal hypertension were recorded, as were comments on liver morphology (e.g., left lobe hypertrophy and nodular liver) and mention of additional abnormalities (splenomegaly, ascites, varices). Imaging data were used to determine maximum tumor size, tumor number, macrovascular invasion (defined as portal vein or hepatic vein thrombus), and presence of metastases at diagnosis. Barcelona Clinic Liver Cancer (BCLC) staging^45^ and Milan criteria^46^ at diagnosis were also determined.

If surgical resection or transplantation were performed, the pathology report was used to obtain the METAVIR score (scale 0 [no fibrosis] to four [cirrhosis]) of the non‐tumor liver parenchyma.^47^ Pathological report data were used to determine tumor size, number, differentiation, microvascular invasion, gross vascular invasion, tumor necrosis, and an American Joint Committee on Cancer (AJCC) 8th edition stage.^48^

### 2.4 Statistical analysis

The Statistical Package for the Social Sciences (SPSS) version 22.0 was used, applying a significance threshold of 0.05. Categorical data were analyzed by Chi square or Fisher's exact tests, continuous variables were analyzed by the Mann–Whitney *U*‐test, survival was analyzed by Kaplan–Meier curves. Multivariable logistic regression analysis was used to identify factors independently associated with HCC beyond Milan criteria at diagnosis; variables with *p* < 0.1 in the univariable analysis were included in the multivariable model.

## 3 RESULTS

### 3.1 HCC patients without HCV exposure

A total of 362 patients did not have a history of HCV exposure: 226 (62.4%) had HBV exposure (with or without HIV exposure), 67 (18.5%) had ALD, and 31 (8.6%) had NAFLD. Of these, 163 individuals (45%) self‐identified as non‐Hispanic Black; the remaining patients self‐identified as non‐Hispanic White (*n* = 119), Hispanic (*n* = 64), and other (*n* = 16). Black and non‐Black individuals did not differ in the distribution of disease etiologies. Black patients were younger than others (median age, inter‐quartile range [IQR] 1–3, 54 (36–63) vs. 60 (52–67), *p* < 0.01) and less likely to have commercial insurance (31.3% vs. 46.7%, *p* < 0.01) (Table 1). The two groups were similar in BMI, the percentage of men, and the prevalence with HIV exposure (Table 1). Among 121 patients who underwent surgery, 80 patients had a liver resection and 41 had a liver transplant; the distribution of the types of surgical procedures was similar in Black and non‐Black patients (Table 1).

**TABLE 1:** Characteristics of HCC patients with HBV, ALD, or NAFLD and no history of HCV exposure.

	Non‐Black	Black	*p*‐value
	*n* = 199	*n* = 163	
Sex, male	157 (78.9%)	121 (74.2%)	0.54
Age (years), median (IQR)	60 (52–67)	54 (46–63)	<0.01
BMI (kg/m^2^), median (IQR)	27 (23.5–30.3)	25.93 (22.5–29.7)	0.55
Commercial insurance	93 (46.7%)	51 (31.3%)	<0.01
HIV co‐infection	35 (17.6%)	25 (15.3%)	0.77

Surgery patients	Non‐Black	Black	
	*n* = 70	*n* = 51	
Resection	45 (64.3%)	35 (68.6%)	0.62
Transplantation	25 (35.7%)	16 (31.4%)	

Abbreviations: ALD, alcohol‐related liver disease; BMI, body mass index; HBV, hepatitis B virus; HCC, hepatocellular carcinoma; HCV, hepatitis C virus; HIV, human immunodeficiency virus; IQR, inter‐quartile range; NAFLD, nonalcoholic fatty liver disease.

Table 2 presents data about the status of the liver at the time of HCC diagnosis. Black patients had less advanced liver disease and were less likely to have indications of portal hypertension or liver decompensation, according to radiology reports (Table 2). Additionally, Black patients had a lower prevalence of ascites and splenomegaly (*p* < 0.05 for both). Black patients had higher median platelet counts (153 x 10^3^/mm^3^ [99–240] vs.139 x 10^3^/mm^3^ [89–187], *p* < 0.01) and a higher percentage had an APRI score below 0.5 (54.6 vs. 42.2, *p* < 0.03). Measurements of bilirubin, INR, and albumin were similar in Black and non‐Black patients. Median FIB‐4 scores were lower in the Black population, but the difference was not statistically significant (3.9 [2.2–6.6] vs. 4.71 [2.4–7.5], *p* = 0.14).

**TABLE 2:** Imaging, laboratory, and histopathological data on HCC patients with HBV, ALD, or NAFLD and no history of HCV exposure.

	Non‐Black	Black	*p*‐value
	*n* = 199	*n* = 163	
Findings based on imaging			
Altered morphology	115 (57.8%)	59 (36.2%)	<0.01
Nodular liver	131 (65.8%)	89 (54.6%)	0.03
Cirrhosis	145 (72.9%)	86 (52.8%)	<0.01
Splenomegaly	89 (44.7%)	37 (22.7%)	<0.01
Ascites	59 (29.6%)	33 (20.2%)	0.04
Varices	77 (38.7%)	54 (33.1%)	0.27
Portal hypertension	107 (53.8%)	52 (31.9%)	<0.01
Findings based on laboratory data			
Child‐Pugh Score			
A	113 (56.8%)	107 (65.6%)	0.04
B	67 (33.7%)	34 (20.9%)	
C	19 (9.5%)	22 (13.5%)	
MELD score, median (IQR)	10 (8–15)	9 (7–17)	0.80
FIB‐4 score, median (IQR)	4.71 (2.4–7.5)	3.9 (2.2–6.6)	0.14
FIB‐4 score < 3.25	67 (33.7%)	67 (41.1%)	0.08
FIB‐4 score < 2.67	55 (27.6%)	54 (33.1%)	0.11
APRI score < 0.5	84 (42.2%)	89 (54.6%)	0.03
Total bilirubin (mg/dL), median (IQR)	1 (0.6–2.3)	0.9 (0.6–2.4)	0.55
INR, median (IQR)	1.2 (1.1–1.4)	1.2 (1.0–1.4)	0.25
Platelets (10^3^/mm^3^), median (IQR)	139 (89–187)	153 (99–240)	<0.01
Albumin (g/dL), median (IQR)	3.6 (3.1–4.1)	3.5 (2.9–4)	0.07

Histopathology of uninvolved liver tissue	Non‐Black	Black	*p*‐value
	*n* = 70	*n* = 52	
Liver fibrosis stage			
1 + 2	8 (11.9%)	8 (19.5%)	0.24
3	8 (11.9%)	8 (19.5%)	
4	51 (76.2%)	25 (61%)	

Abbreviations: ALD, alcohol‐related liver disease; APRI, AST to platelet ratio index; FIB‐4 score, fibrosis‐4 score; HBV, hepatitis B virus; HCC, hepatocellular carcinoma; HCV, hepatitis C virus; INR, international normalized ratio; IQR, inter‐quartile range; MELD, model end‐stage liver disease; NAFLD, nonalcoholic fatty liver disease.

**FIGURE 1:**
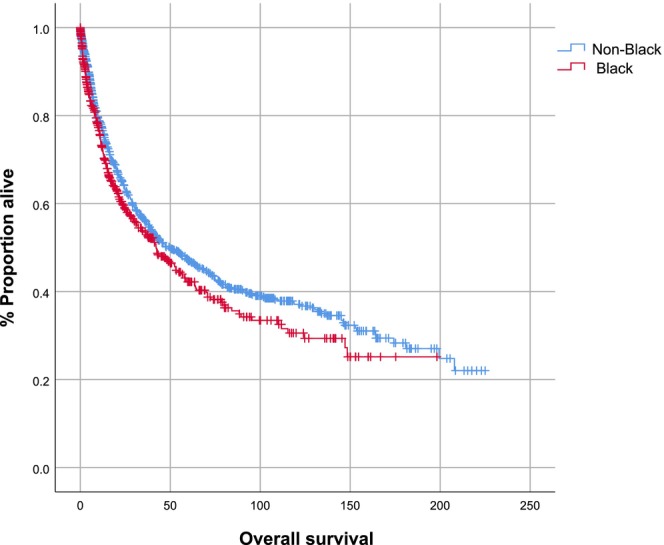
Kaplan–Meier analysis of overall survival of Black versus non‐Black patients with chronic HBV infection, ALD, or NAFLD as the underlying liver disease. The graph shows the proportion of surviving patients. HBV, hepatitis B virus; ALD, alcohol‐associated liver disease; NAFLD, non‐alcoholic fatty liver disease. Black patients (red line); other patients (blue line). The p value of the comparison is 0.17.

Although the underlying liver disease was less advanced, Black individuals had more advanced HCC and were less likely to have curable liver cancer. The imaging data in Table 3 show that Black patients had larger tumors (median, IQR [1–3], 4.3 [2.3–10.4] cm vs. 3.6 [2.3–6.3] cm, *p* < 0.01), more gross vascular invasion (thrombus in portal vein or hepatic vein) (38.0% vs. 23.1%, *p* < 0.01), and more metastatic disease (14.7% vs. 7.0%, *p* = 0.02). A smaller percentage of HCC in Black patients was within Milan criteria: 35.6% versus 48.7%, *p* = 0.01. According to BCLC staging, Black patients had more advanced cancer than those who were not Black (*p* < 0.01), with a smaller percentage of stage A, which represents disease that is more likely to be curable: 34.4% versus 55.3%, *p* < 0.01. Among patients who underwent surgery (Table 3), histopathology revealed that Black patients had a higher percentage of poorly differentiated tumors (32.7% vs. 22.9%, *p* = 0.05) and a higher percentage of tumors with microvascular invasion (76.9% vs. 60%, *p* = 0.04). Fewer Black patients presented with early stage (T1) disease (15.4% vs. 40.0%, *p* < 0.01). Median follow up was 13 months (IQR [1–3], 2–4). Black patients had shorter median survival (median, IQR [1–3], 10 [2–32] months vs. 13 [4–52] months, *p* = 0.03], although survival did not differ significantly by Kaplan–Meier analysis (*p* = 0.17) (Figure 1).

**TABLE 3:** Tumor characteristics by imaging, AFP measurement, and histopathology in HCC patients with HBV, ALD, or NAFLD without a history of HCV exposure.

	Non‐Black	Black	*p*‐value
	*n* = 199	*n* = 163	
Imaging and AFP data			
Size of largest tumor (cm), median (IQR)	3.6 (2.3–6.3)	4.3 (2.3–10.4)	<0.01
Number of tumors, median (IQR)	1 (1–1)	1 (1–1)	0.78
Gross vascular invasion	46 (23.1%)	62 (38.0%)	<0.01
Metastases	14 (7.0%)	24 (14.7%)	0.02
AFP (ng/mL), median (IQR)	24 (5–678)	198 (14–7982)	<0.01
Within Milan criteria	97 (48.7%)	58 (35.6%)	0.01
BCLC criteria			
A	110 (55.3%)	56 (34.4%)	<0.01
B	27 (13.6%)	26 (16%)	
C	44 (22.1%)	59 (36.2%)	
D	18 (9%)	22 (13.5%)	

Tumor histopathology	Non‐Black	Black	*p*‐value
	*n* = 70	*n* = 52	
Tumor size (cm), median (IQR)	3.4 (2.3–6.0)	3.6 (2.5–6.6)	0.62
Number of tumors, median (IQR)	1 (1–1)	1 (1–2)	0.11
Microvascular invasion	42 (60.0%)	40 (76.9%)	0.04
Gross vascular invasion	11 (15.7%)	3 (5.8%)	0.70
Poor differentiation	16 (22.9%)	17 (32.7%)	0.05
Tumor necrosis	8 (11.4%)	5 (9.6%)	0.57
AJCC stage			
Stage 1	28 (40.0%)	8 (15.4%)	0.02
Stage 2	38 (54.3%)	33 (63.5%)	
Stage 3 + 4	4 (5.7%)	11 (21.1%)	

Abbreviations: AFP, alpha fetoprotein; AJCC, American Joint Committee on Cancer; ALD, alcohol‐related liver disease; BCLC, Barcelona Clinic Liver Cancer; HBV, hepatitis B virus; HCC, hepatocellular carcinoma; IQR, inter‐quartile range; NAFLD, non‐alcoholic fatty liver disease.

### 3.2 HCC in patients with liver diseases of all major etiologies: HCV, HBV, ALD, and NAFLD

**FIGURE 2:**
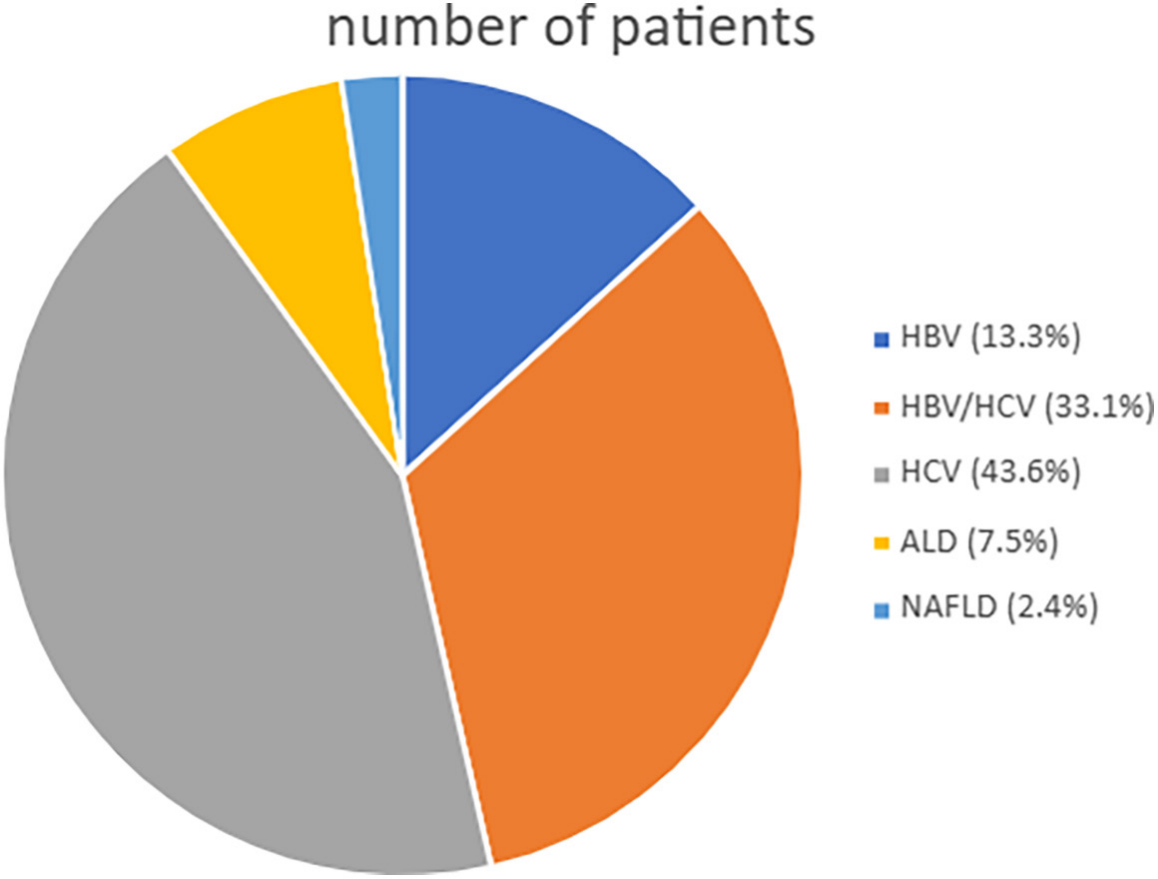
The distribution of underlying liver diseases in the combined cohort of patients with chronic HCV infection, chronic HBV infection, ALD or MASLD. HCV, hepatitis C virus; HBV, hepatitis B virus; ALD, alcohol‐associated liver disease; NAFLD, non‐alcoholic  fatty liver disease. The sizes of the sectors in the pie chart denote the percentage of the study cohort with each underlying liver disease.

We analyzed a cohort of 1557 patients with HCC, which included patients with HCV exposure whom we analyzed previously.^18^ Figure 2 shows the distribution of the underlying liver diseases. The cohort included 411 non‐cirrhotic patients. The age distribution and percentage of men did not differ between Black (*n* = 553) and non‐Black (*n* = 1004) patients' groups (Table 4). HBV infection (with or without HCV co‐infection) was more prevalent in Black patients than in others: 56.4% versus 40.9%, *p* < 0.01. A lower percentage of Black patients had commercial insurance: 27.5% versus 41.7%, *p* < 0.01, and a higher percentage had a FIB‐4 score below 3.25: 34.4% versus 20.9%, *p* < 0.01. Black individuals had better liver function and less cirrhosis at the time of HCC diagnosis, according to laboratory, radiologic, and histopathologic data (Table 4).

**TABLE 4:** Characteristics and liver function at HCC diagnosis in patients with HCV, HBV, ALD, or NAFLD.

	Non‐Black	Black	*p*‐value
	*n* = 1004	*n* = 553	
Demographic and clinical characteristics			
Sex, male	778 (77.5%)	409 (73.9%)	0.19
Age (years), median (IQR)	59 (54–67)	60 (57–66)	0.12
BMI (kg/m2), median (IQR)	26.84 (23.8–30.2)	26.25 (22.62–29.6)	0.02
Commercial Insurance	419 (41.7%)	152 (27.5%)	<0.01
Underlying liver disease			
HBV	107 (10.6%)	100 (18.1%)	<0.01
HCV	501 (49.9%)	178 (32.2%)	<0.01
HBV/HCV	304 (30.3%)	212 (38.3%)	<0.01
ALD	68 (6.8%)	49 (8.9%)	0.75
NAFLD	24 (2.4%)	14 (2.5%)	0.71
Laboratory criteria			
Child Pugh Score			
A	582 (58.1%)	378 (68.4%)	0.01
B	296 (29.4%)	130 (23.5%)	
C	126 (12.5%)	45 (8.1%)	
MELD score, median (IQR)	10 (7–16)	9 (7–15)	0.09
FIB‐4 score, median (IQR)	6.14 (3.6–9.8)	4.38 (2.70–7.4)	<0.01
FIB‐4 score < 3.25	210 (20.9%)	190 (34.4%)	<0.01
FiB‐4 score < 2.67	156 (15.5%)	135 (24.4%)	<0.01
APRI score	0.81 (0.4–1.5)	0.54 (0.30–1.1)	<0.01
Total bilirubin (mg/dL), median (IQR)	1.1 (0.7–2.2)	0.9 (0.6–1.7)	<0.01
INR, median (IQR)	1.2 (1.1–1.4)	1.1 (1.0–1.3)	<0.01
Platelets (10^3^/mm^3^), median (IQR)	110 (73–164)	147 (100–205)	<0.01
Albumin (g/dL), median (IQR)	3.4 (2.9–3.9)	3.4 (2.9–3.9)	0.33
Findings based on imaging			
Change in morphology	528 (52.6%)	216 (39.0%)	<0.01
Nodular liver	729 (72.6%)	279 (50.5%)	<0.01
Cirrhosis	747 (74.4%)	270 (48.8%)	<0.01
Ascites	310 (30.9%)	105 (18.9%)	<0.01
Splenomegaly	514 (51.2%)	105 (18.9%)	<0.01
Varices	461 (45.9%)	129 (23.3%)	<0.01
Portal hypertension	522 (51.9%)	130 (23.5%)	<0.01

Histopathology of uninvolved liver tissue	Non‐Black	Black	*p*‐value
	*n* = 300	*n* = 147	
Liver fibrosis stage			
Stage 1 + 2	29 (9.7%)	31 (21.1%)	<0.01
Stage 3	33 (11.1%)	23 (15.6%)	
Stage 4	238 (79.2%)	93 (63.3%)	

Abbreviations: ALD, alcohol associated liver disease; APRI, AST to platelet ratio index; BMI, body mass index; FIB‐4 score, fibrosis‐4 score; HBV, hepatitis B virus; HCV, hepatitis C virus; HCC, hepatocellular carcinoma; INR, international normalized ratio; IQR, inter‐quartile range; MELD, model end‐stage liver disease; NAFLD, non‐alcoholic fatty liver disease.

Patients with and without cirrhosis were compared to each other as a total cohort and after stratification by race/ethnicity. Patients with and without cirrhosis did not differ by age. Black patients without cirrhosis were more likely to have commercial insurance than Black patients with cirrhosis, although the percentages were low in both groups (34.7% vs. 23.7%, *p* < 0.01). Non‐Black patients without cirrhosis were more likely to be male than non‐Black patients with cirrhosis (80.5% vs. 67.3% *p* < 0.01).

**FIGURE 3:**
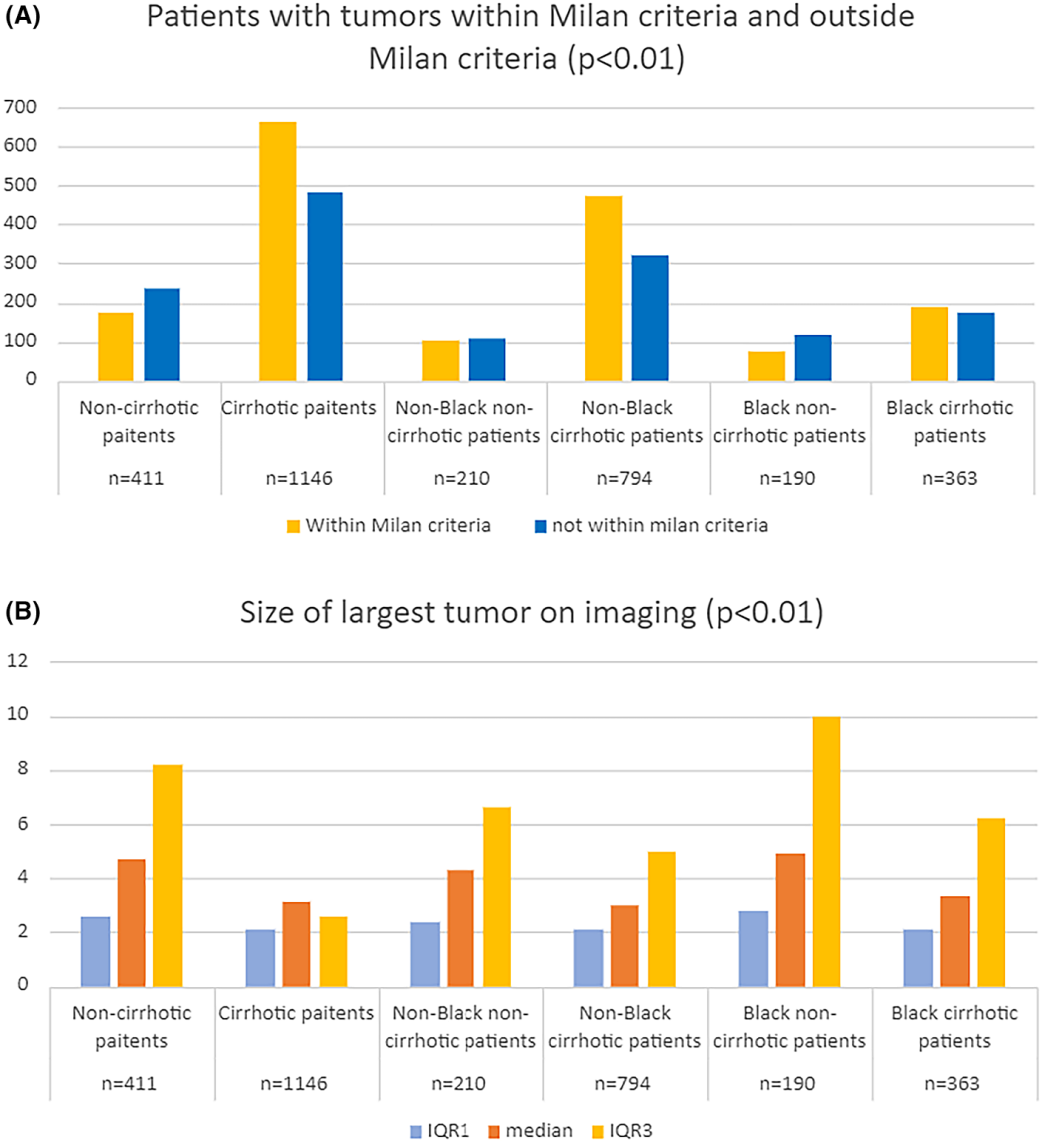
The number of patients within Milan criteria and tumor size. (A) Bar graphs show the number of patients with hepatitis C virus (HCC) within (gold) or beyond (blue) Milan criteria. (B) Bar graphs show the median (orange) and interquartile (IQR) ranges 1 (blue) and 3 (gold) of the sizes of the HCCs as determined by imaging.

**FIGURE 4:**
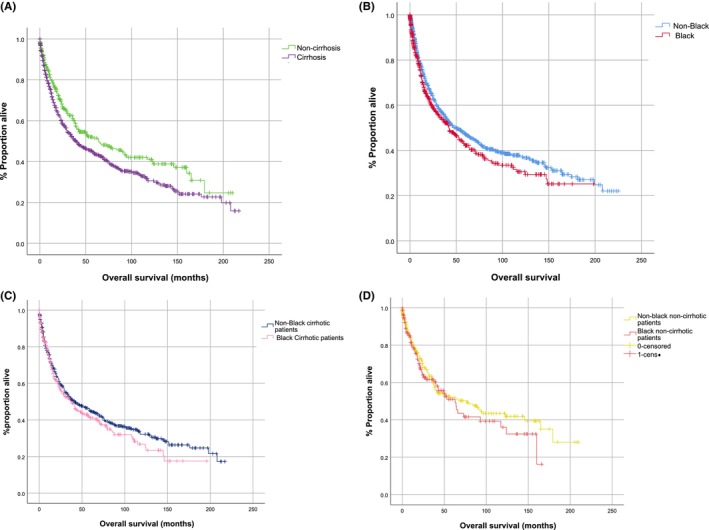
Kaplan–Meier analysis of overall survival of the combined cohort of patients with chronic HCV infection, chronic HBV infection, ALD or NAFLD. Graph (A) shows the comparison of survival in cirrhotic versus non‐cirrhotic patients; *p* = 0.03. Graph (B) shows the comparison of survival in Black versus non‐Black patients; *p* < 0.01. Graph (C) shows the comparison of survival in Black versus non‐Black patients with cirrhosis; *p* = 0.43. Graph (D) shows the comparison of survival of Black versus non‐Black patients without cirrhosis; the *p* = 0.19. HCV, hepatitis C virus; HBV, hepatitis B virus; ALD, alcohol‐associated liver disease; NAFLD, non‐alcoholic fatty liver disease.

Tumors of non‐cirrhotic patients had worse features than those of cirrhotic patients in the cohort as a whole, and in Black and non‐Black groups analyzed separately (Table 5). Non‐cirrhotic patients were less likely to be within Milan criteria (42.6% vs. 57.7%, *p* < 0.01) (Table 5; Figure 3A) and had larger tumors on imaging (4.7 vs. 3.1 cm, *p* < 0.01) (Table 5; Figure 3B). A total of 517 patients (194 without cirrhosis and 323 with cirrhosis) had liver resection or liver transplantation and had a pathology report available. A higher percentage of tumors in non‐cirrhotic patients were poorly differentiated (28.9% vs. 21.4%, *p* = 0.05) and showed microvascular invasion (67.0% vs. 56.8%, *p* = 0.02). Despite many adverse prognostic features of their tumors, patients without cirrhosis had better overall survival (median, IQR [1–3], 21 [4–51] months vs. 17 [5–57] months, *p* < 0.01), likely because of their underlying liver disease was less advanced (Figure 4A). Median follow up was 29 months.

**TABLE 5:** HCC patients with HCV, HBV, ALD or NAFLD stratified by the presence or absence of cirrhosis.

	All patients			Black patients			Non‐Black patients		
	Cirrhotic	Non‐cirrhotic	*p*‐value	Cirrhotic	Non‐cirrhotic	*p*‐value	Cirrhotic	Non‐cirrhotic	*p*‐value
	*n* = 1146	*n* = 411		*n* = 363	*n* = 190		*n* = 794	*n* = 210	
Sex, male	808 (70.5%)	311 (75.7%)	0.05	274 (75.5%)	142 (74.7%)	0.85	534 (67.3%)	169 (80.5%)	<0.01
Age (years), median (IQR)	60 (54–66)	60 (54–66)	0.08	60 (55–66)	60 (52–66)	0.23	60 (54–67)	60 (54–66)	0.28
Commercial Insurance	407 (35.5%)	164 (39.9%)	0.12	86 (23.7%)	66 (34.7%)	<0.01	321 (40.4%)	98 (46.6%)	0.10
Size of largest tumor (cm), median (IQR)	3.1 (2.1–5.3)	4.7 (2.6–8.2)	<0.01	3.3 (2.1–6.2)	4.9 (2.8–10)	<0.01	3 (2.1–5.0)	4.3 (2.4–6.6)	<0.01
Number of tumors, median (IQR)	1 (1–2)	1 (1–2)	0.06	1 (1–3)	1 (1–2)	0.24	1 (1–2)	1 (1–2)	0.33
Gross vascular invasion	244 (21.3%)	93 (22.6%)	0.30	94 (25.9%)	50 (26.3%)	0.93	150 (18.9%)	43 (20.5%)	0.94
Metastases	90 (7.9%)	41 (9.9%)	0.25	39 (10.7%)	25 (13.1%)	0.44	51 (6.4%)	16 (7.6%)	0.69
Within Milan criteria	661 (57.7%)	175 (42.6%)	<0.01	190 (52.3%)	74 (38.9%)	<0.01	471 (59.3%)	101 (48.1%)	<0.01

	All patients			Black patients			Non‐Black patients		
	Cirrhotic	Non‐cirrhotic	*p*‐value	Cirrhotic	Non‐cirrhotic	*p*‐value	Cirrhotic	Non‐cirrhotic	*p*‐value
							*n* = 228	*n* = 118	
	*n* = 323	*n* = 194		*n* = 95	*n* = 76				
Size of largest tumor (cm), median (IQR)	3.0 (2.0–4.1)	4.0 (2.5–6.5)	0.42	3.0 (2.0–4.4)	4.5 (2.7–7.0)	<0.01	3.0 (2.0–4.0)	3.5 (2.3–6)	<0.01
Number of tumors, median (IQR)	1 (1–2)	1 (1–2)	0.14	1 (1–3)	1 (1–1)	<0.01	1 (1–2)	1 (1–1)	<0.01
Microvascular invasion	183 (56.8%)	130 (67.0%)	0.02	66 (69.4%)	54 (71.1%)	0.97	117 (51.3%)	76 (64.4%)	<0.01
Poor differentiation	69 (21.4%)	56 (28.9%)	0.05	24 (25.2%)	29 (38.2%)	0.09	45 (19.7%)	27 (22.9%)	0.47
AJCC stage									
Stage 1	102 (31.6%)	47 (24.2%)	<0.01	22 (23.1%)	10 (13.1%)	<0.01	80 (35.1%)	37 (31.4%)	<0.01
Stage 2	190 (58.8%)	99 (51.0%)		62 (65.3%)	42 (55.3%)		128 (56.1%)	57 (48.3%)	
Stage 3 + 4	31 (9.6%)	48 (24.8%)		11 (11.6%)	24 (31.6%)		20 (8.8%)	24 (20.3%)	

Abbreviations: AJCC, American Joint Committee on Cancer; ALD, alcohol‐related liver disease; HBV, hepatitis B virus; HCC, hepatocellular carcinoma; HCV, hepatitis C virus; IQR, inter‐quartile range; NAFLD, non‐alcoholic fatty liver disease.

A comparison of Black and non‐Black patients showed that Black patients presented with more advanced cancer as determined by both radiologic and pathologic criteria (Table 6). They had larger tumors on imaging (3.8 cm vs. 3.3 cm, *p* < 0.01) and had more gross vascular invasion (144 [27.1%] vs. 193 [19.2%], *p* = 0.03). Black patients had more distant metastases (64 [12.0%] vs. 67 [6.7%], *p* = 0.01), and were less likely to be within Milan criteria for transplant eligibility (264 [47.7%] vs. 572 [56.9%], *p* < 0.01). Of the 517 patients with pathology reports, Black patients had less early‐stage HCC (18.7% vs. 33.8%, *p* < 0.01); larger tumors (3.5 cm vs. 3.0 cm), although the difference was not statistically significant (*p* = 0.07); a higher percentage of poorly differentiated tumors (31.0% vs. 20.8%, *p* = 0.01); and more microvascular invasion (70.2% vs. 55.8%, *p* < 0.01). Black patients were less likely to have AJCC Stage 1 disease (32 [18.7%] vs. 117 [33.8%]) and more likely to have Stage 3 or 4 disease (35 [20.5%] vs. 44 [12.7%], *p* < 0.01). Black patients also had higher median AFP levels (59.6 vs. 32.4 ng/mL, *p* < 0.01). Black patients had significantly shorter overall median survival: 16 months versus 21 months, *p* < 0.01 (Figure 4B). Because HCC had worse characteristics in non‐cirrhotic patients of all racial/ethnic groups (compared to cirrhotic patients), additional subgroup analyses were performed to further investigate the relationships between tumor characteristics and race/ethnicity on the one hand, and tumor characteristics and the absence of cirrhosis on the other.

**TABLE 6:** HCC patients with HCV, HBV, ALD or NAFLD stratified by the presence or absence of cirrhosis and by race/ethnicity (Black vs. non‐Black patients).

	All patients			Cirrhotic patients			Non‐cirrhotic patients		
	Black	Non‐Black	*p*‐value	Black	Non‐Black	*p*‐value	Black	Non‐Black	*p*‐value
	*n* = 553	*n* = 1004		*n* = 363	*n* = 794		*n* = 190	*n* = 210	
Sex (male)	409 (73.9%)	778 (77.5%)	0.19	274 (75.5%)	534 (67.3%)	<0.01	142 (74.7%)	169 (80.5%)	0.17
Age (years), median (IQR)	59 (54–67)	60 (54–66)	0.97	60 (54–67)	60 (55–66)	0.95	60 (53–66)	59 (53–65)	0.54
Commercial insurance	152 (28.7%)	419 (42.8%)	<0.01	86 (23.7%)	321 (40.4%)	<0.01	66 (34.7%)	98 (46.6%)	<0.01

				Black	Non‐Black	*p*‐value	Black	Non‐Black	*p*‐value
				*n* = 363	*n* = 794		*n* = 190	*n* = 210	
Size of largest tumor (cm), median (IQR)	3.8 (2.3–7.4)	3.3 (2.1–5.3)	<0.01	3.3 (2.1–6.2)	3.0 (2.1–5.0)	0.03	4.9 (2.8–10)	4.3 (2.4–6.6)	<0.01
Number of tumors, median (IQR)	1 (1–3)	1 (1–2)	<0.01	1 (1–3)	1 (1–2)	0.08	1 (1–2)	1 (1–2)	0.30
Gross vascular invasion	144 (27.1%)	193 (19.2%)	0.03	94 (25.9%)	150 (18.9%)	<0.01	50 (26.3%)	43 (20.5%)	0.16
Metastases	64 (12.0%)	67 (6.7%)	0.01	39 (10.7%)	51 (6.4%)	0.01	25 (13.1%)	16 (7.6%)	0.06
Within Milan criteria	264 (47.7%)	572 (56.9%)	<0.01	190 (52.3%)	471 (59.3%)	0.03	74 (38.9%)	101 (48.1%)	0.06

	All patients			Cirrhotic patients			Non‐cirrhotic patients		
	Black	Non‐Black	*p*‐value	Black	Non‐Black	*p*‐value	Black	Non‐Black	*p*‐value
	*n* = 171	*n* = 346		*n* = 95	*n* = 228		*n* = 76	*n* = 118	
Size of largest tumor (cm), median (IQR)	3.5 (2.3–6.0)	3.0 (2.1–4.5)	0.07	3 (2.0–4.4)	3.0 (2.0–4.0)	0.75	4.5 (2.7–7.0)	3.5 (2.3–6)	0.05
Number of tumors, median (IQR)	1 (1–2)	1 (1–2)	0.13	1 (1–3)	1 (1–2)	0.02	1 (1–1)	1 (1–1)	0.72
Microvascular invasion	120 (70.2%)	193 (55.8%)	<0.01	66 (69.4%)	117 (51.3%)	<0.01	54 (71.1%)	76 (64.4%)	0.38
Poor differentiation	53 (31.0%)	72 (20.8%)	0.01	24 (25.2%)	45 (19.7%)	0.27	29 (38.2%)	27 (22.9%)	0.02
AJCC stage									
Stage 1	32 (18.7%)	117 (33.8%)	<0.01	22 (23.1%)	80 (35.1%)	0.10	10 (13.1%)	37 (31.4%)	0.01
Stage 2	104 (60.8%)	185 (53.5%)		62 (65.3%)	128 (56.1%)		42 (55.3%)	57 (48.3%)	
Stage 3 + 4	35 (20.5%)	44 (12.7%)		11 (11.6%)	20 (8.8%)		24 (31.6%)	24 (20.3%)	

Abbreviations: AJCC, American Joint Committee on Cancer; ALD, alcohol‐related liver disease; HBV, hepatitis B virus; HCC, hepatocellular carcinoma; HCV, hepatitis C virus; IQR, inter‐quartile range; NAFLD, non‐alcoholic fatty liver disease.

In a comparison limited to patients with cirrhosis (Table 6), Black patients had larger tumors (3.3 cm vs. 3.0 cm, *p* = 0.03); a higher percentage of tumors showing gross vascular invasion (25.9% vs. 18.9%, *p* < 0.01); and more frequent distant metastases (10.7% vs. 6.4%, *p* = 0.01). They were also less likely to be within Milan criteria (52.3% vs. 59.3%, *p* =0.03). On histopathology, a higher percentage of their HCCs showed microvascular invasion (69.4% vs. 51.3%, *p* < 0.01). Black patients tended to have shorter overall survival, but the difference was not statistically significant, likely due to the smaller sample size in this subset analysis (Figure 4C). In a parallel comparison limited to patients without cirrhosis, Black patients had larger tumors (4.9 vs. 4.3 cm, *p* < 0.01), more frequent distant metastases (13.1% vs. 7.6%) and a lower percentage within Milan criteria (38.9% vs. 48.1%, although the latter two findings did not reach statistical significance (*p* = 0.06 for both).

In a comparison limited to Black patients, those without cirrhosis had larger tumors on imaging (4.9 cm vs. 3.3 cm, *p* < 0.01) and on pathology (4.5 cm vs. 3.0 cm, *p* < 0.01) and they were less likely to be within Milan criteria (38.9% vs. 52.3%, *p* < 0.01); however, HCC histologic grade did not differ. In a comparison limited to non‐Black individuals, non‐cirrhotic patients had larger tumors (4.3 cm vs. 3.0 cm, *p* < 0.01), were less likely to be within Milan criteria (48.1% vs. 59.3%) and had more microvascular invasion (64.4% vs. 51.3%, *p* < 0.01), but histologic grade did not differ (Table 6). Black patients without cirrhosis tended to have worse survival than other non‐cirrhotic patients, but the difference was not statistically significant (Figure 4D).

Because a patient's status with respect to the Milan criteria may determine eligibility for liver transplantation and thus overall survival, we chose this variable as the best representation of favorable tumor characteristics at the time of HCC diagnosis. Logistic regression was performed to identify factors independently associated with failure to meet Milan criteria (Table 7). In both univariable and multivariable analysis, the lack of commercial insurance (OR 1.45 [CI 95% 1.19–1.83], *p* < 0.01), male sex (OR 1.34 [CI 95% 1.05–1.70], *p* < 0.01), absence of cirrhosis (OR 1.58 [CI 95% 1.27–1.98], *p* 0.01) and Black race/ethnicity (OR 1.34 [CI 95% 1.09–1.66], *p* = 0.01) were associated with tumor characteristics beyond Milan criteria. Neither age, nor HBV exposure was associated with tumor burden beyond Milan criteria.

**TABLE 7:** Univariable and multivariable logistic regression analysis of factors associated with having HCC outside Milan criteria at diagnosis.

	Univariable analysis			Multivariable analysis		
	Odds ratio	95% Confidence interval	*p*‐value	Odds ratio	95% Confidence interval	*p*‐value
Male	1.32	1.04–1.67	0.03	1.34	1.05–1.70	0.02
Age	0.99	0.98–1.00	0.11			
Lack of commercial insurance	1.51	1.22–1.87	<0.01	1.45	1.19–1.83	<0.01
BMI	0.98	0.97–1.01	0.73			
FIB‐4 score <3.25	1.65	1.32–2.05	<0.01	1.58	1.27–1.98	<0.01
Black race	1.44	1.17–1.78	<0.01	1.34	1.09–1.66	0.01
HBV exposure	1.12	0.92–1.37	0.48			

Abbreviations: BMI, body mass index; FIB‐4 score, fibrosis‐4 score; HBV, hepatitis B virus; HCC, hepatocellular carcinoma.

## 4 DISCUSSION

This analysis of 1557 patients with HCC who had a variety of chronic liver diseases (HCV, HBV, ALD, and NAFLD), had three notable findings. First, we found that HCC in non‐cirrhotic patients has worse prognostic features than HCC in cirrhotic patients, irrespective of the underlying liver disease, as expected. This finding is supported by data from Martino et al.,^49^ who also found that HCC lesions in non‐cirrhotic patients were significantly larger than lesions in cirrhotic patients. In a study by Gawrieh et al.,^50^ non‐cirrhotic patients with HCC presented with larger and more advanced HCC that was more commonly outside the Milan criteria. Interestingly, a recent molecular analysis of HCC in patients with NAFLD by Pinyol et al.,^51^ revealed that the tumors in non‐cirrhotic patients had a higher mutational burden than tumors in cirrhotic patients; this higher mutational burden could underlie a more aggressive tumor biology and a different pathway of mutagenesis and tumorigenesis.

Second, we found that Black patients have more advanced HCC, as evidenced by larger tumors, a higher percentage of poorly differentiated tumors, tumors with gross and microscopic vascular invasion, higher AFP levels, and a higher prevalence of metastatic disease, as we hypothesized. These patients also had higher mortality, consistent with prior reports that Black patients with HCC have shorter survival than non‐Black patients.^16^, ^18^, ^26^ In our study, Black patients were less likely to have commercial insurance than non‐Black patients, raising the possibility that barriers to accessing healthcare may contribute to diagnostic delays.

Third, our analysis of 1557 patients with most major types of chronic liver disease and our sub‐analysis of 362 patients with no history of HCV exposure confirm and extend previous evidence^18^, ^19^, ^20^, ^21^ that Black patients have less advanced liver disease at the time of HCC diagnosis than patients of other racial/ethnic groups. Multivariable logistic regression showed that lack of private insurance, a FIB‐4 score below 3.25 (indicating the absence of cirrhosis), male sex, and Black race increased the odds that a patient would present with HCC outside Milan criteria, limiting treatment options. Interestingly, HBV exposure, as indicated by serology, did not increase the odds.

Our observation that Black patients were more likely to develop HCC in the absence of cirrhosis differs from the findings of Gawrieh et al.,^50^ who studied 605 non‐cirrhotic patients with HCC and determined that Black patients were less likely to develop HCC in non‐cirrhotic liver than members of other racial/ethnic groups. The discrepancy is likely to reflect differences in the distribution of the chronic liver diseases in the two studies. In their study, 42.5% of the non‐cirrhotic patients had an unclear or unknown underlying liver disease etiology, whereas our study required evidence of exposure to viral hepatitis or a diagnosis of ALD or NAFLD. Their high prevalence of non‐cirrhotic HCC patients whose underlying liver disease etiology was unknown is striking and suggests a need for research to identify additional HCC risk factors. In our study all non‐cirrhotic patients had a FIB‐4 score below 3.25; whereas in the study by Gawrieh and colleagues, 20.4% of patients in the non‐cirrhotic group had a FIB‐4 score above 3.6. Because histopathology data were often lacking, we felt using a uniform standard, the FIB‐4 score, was the most reliable method for distinguishing cirrhotic and non‐cirrhotic patients because it could be applied to all patients; however, we acknowledge the limitation of using non‐invasive markers to stage fibrosis. Previous studies have shown that for HCV‐infected patients, FIB‐4 below 3.25 has a specificity of 98.2% to rule out fibrosis,^39^ and for patients with NAFLD, FIB‐4 below 3.25 has a specificity of 95.8%.^52^ In our study, imaging and histopathology data strongly support the conclusion that liver fibrosis and cirrhosis were less advanced in Black patients with HCC.

We propose that the tendency of Black patients to develop HCC in the absence of advanced cirrhosis contributes to their higher mortality^53^ by reducing the likelihood that HCC will be detected at an early and curable stage through HCC surveillance, which is primarily directed toward patients with cirrhosis^11^, ^12^, ^13^, ^14^, ^15^ While the greatest reduction in HCC mortality would come from reducing rates of chronic liver disease (e.g., viral hepatitis, alcohol, smoking and toxic exposures, and metabolic dysfunction), progress toward eliminating disparities might be achieved through expanded health insurance coverage, the development of new methods for detecting HCC and for identifying high risk non‐cirrhotic patients.

The need to modify surveillance guidelines so that they provide equitable access has been discussed in the lung cancer field in light of data showing that a significantly lower percentage of African American smokers qualify for surveillance than white smokers.^54^ Despite smoking fewer cigarettes per day and having a lower pack‐year history, African American smokers have a higher risk of lung cancer compared with White smokers, particularly among men. According to the authors, a reduction to 20 pack‐years for African American smokers, as opposed to the current 30 pack‐year threshold, would put African American smokers on par with White smokers. An analogous modification may be needed to ensure that Black and non‐Black patients with a similar level of HCC risk have the same probability of qualifying for HCC surveillance.

Limitations of our study include the retrospective design and enrollment at a single site, which can lead to bias and limited generalizability. Second, cirrhosis was defined by the FIB‐4 score, which has a high specificity, but low sensitivity, meaning that many patients with cirrhosis have FIB‐4 scores below the threshold used in our investigation.^42^ Third, 90% of the study group had a history of viral hepatitis, only 7.5% had ALD and only 2.4% had NAFLD. As improvements in HCV and HBV management are made, viral hepatitis will decline as an HCC risk factor and the type of analysis presented here will need to be repeated. Fourth, HBV‐exposed Asian patients were excluded due to the increased likelihood that they acquired HBV through vertical transmission. Recent data show that HBV integrates into host chromosomes during the initial stage of infection,^37^ suggesting that the prolonged exposure to HBV which typically results from vertical transmission carries a significantly different level of risk and mode of carcinogenesis than horizontal transmission. Thus, including Asian HBV patients would have been a confounder in the analysis.

In conclusion, HCC in non‐cirrhotic patients tends to be diagnosed at a later stage than HCC in cirrhotic patients. Black patients are more likely to develop HCC in non‐cirrhotic liver than other patients and thus, are disproportionately affected by this tendency. Overall, non‐cirrhotic patients have longer survival than cirrhotic patients, presumably because their better liver function more than compensates for their more advanced HCC. However, despite having better liver function at the time of HCC diagnosis, Black patients experience reduced survival. This paradox needs to be resolved through additional research to delineate the contributions of tumor biology and socioeconomic factors and to develop diagnostic tests to identify high‐risk non‐cirrhotic patients who would benefit from regular HCC surveillance.

## AUTHOR CONTRIBUTIONS

**Tali Shaltiel:** Data curation (equal); formal analysis (equal); writing – original draft (equal); writing – review and editing (equal). **Umut Sarpel:** Conceptualization (equal); writing – original draft (equal); writing – review and editing (equal). **Andrea D. Branch:** Conceptualization (equal); formal analysis (equal); writing – original draft (equal); writing – review and editing (equal).

## FUNDING INFORMATION

Supported in part by the Prevent Cancer Foundation, U01OH012263, and U01 OH012622 from the National Institute for Occupational Safety and Health and U01CA288425 to ADB and 1R03CA164546‐01A1 from the National Cancer Institute to US.

## CONFLICT OF INTEREST STATEMENT

Mount Sinai receives funds from Boehringer Ingelheim and Gilead Sciences to support research in Dr. Branch's laboratory.

## Data Availability

The data that support the findings of this study are available from the corresponding author upon reasonable request.
