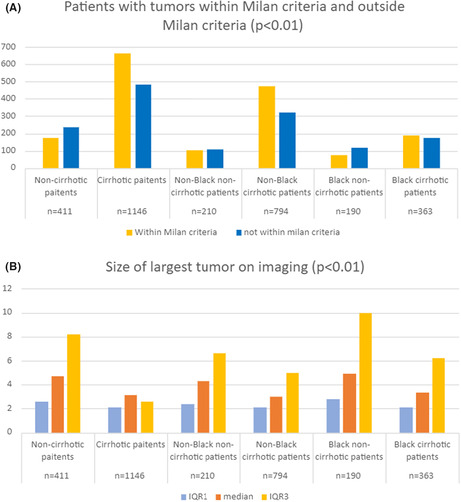# Correction to “The adverse characteristics of hepatocellular carcinoma in the non‐cirrhotic liver disproportionately disadvantage black patients”

**DOI:** 10.1002/cam4.7099

**Published:** 2024-04-24

Shaltiel T, Sarpel U, Branch AD. The adverse characteristics of hepatocellular carcinoma in the non‐cirrhotic liver disproportionately disadvantage Black patients. *Cancer Med*. 2024;13:e6654. doi:10.1002/cam4.6654

A revised Figure 3 is shown below and has been added to the online version of the article.